# A case report of anaplastic thyroid cancer and papillary thyroid cancer lymph node metastasis: an unusual presentation as an invasive hypopharyngeal mass

**DOI:** 10.1259/bjrcr.20210236

**Published:** 2021-12-24

**Authors:** Veena Vishwanath, Nichola Gaunt, Durgesh Rana, Dominic St Leger, Michael Dykes, Weihong Ma, Kate Garcez, Navin Mani

**Affiliations:** 1 Manchester University NHS Trust, England, United Kingdom

## Abstract

Anaplastic thyroid carcinoma is a rare undifferentiated tumour of the thyroid follicular epithelium. It almost always develops from a pre-existing well-differentiated thyroid cancer with a co-existent thyroid malignancy varying from 5 to 17% . The co-existence of papillary thyroid cancer (PTC) with anaplastic thyroid cancer is a rare occurrence in metastases outside the primary thyroid lesion. Traditionally, this has been regarded as an aggressive form of cancer associated with a dismal prognosis. Recently, the focus has shifted to the development of novel therapies based on the availability of comprehensive genomic profiling platforms (CGP) with a rapid turn-around to identify molecular aberrations in tumours which acts as potential therapeutic targets. In the United Kingdom, we report the case of a 60-year-old woman with an unusual presentation of (metastatic) anaplastic thyroid carcinoma and concomitant papillary thyroid cancer metastasis within a contralateral lymph node. This was initially perceived as a left pyriform fossa mass involving and compressing her left hemi-larynx on clinical and radiological examination. Following the identification of BRAF V600E mutation on CGP, she was started on targeted therapy with the BRAF inhibitor dabrafenib and the MEK inhibitor trametinib and demonstrated excellent clinical and radiological response following 7 months of treatment. She has subsequently undergone total thyroidectomy alongside with bilateral neck dissection, and is due to start radioactive iodine treatment to reduce the risk of recurrence of disease.

## Background

Anaplastic thyroid carcinoma is a rare undifferentiated tumour of the thyroid follicular epithelium, with an age adjusted annual incidence of one–two per million persons worldwide.^1–3^ It almost always develops from a pre-existing well-differentiated thyroid cancer^4^ with a co-existent thyroid malignancy varying from 5 to 17%.^5^Traditionally, this has been regarded as an aggressive form of cancer associated with a dismal prognosis. Average patient 5-year survival rates are estimated at approximately 7%, with a median survival time of 6 months from diagnosis.^6,7^ Recently, the focus has shifted to the development of novel therapies based on the availability of comprehensive genomic profiling platforms (CGP) with a rapid turn-around to identify molecular aberrations in tumours which acts as potential therapeutic targets.

The co-existence of papillary thyroid cancer (PTC) with anaplastic thyroid cancer is a rare occurrence in metastases outside the primary thyroid lesion. In the United Kingdom, we report the case of a 60-year-old female with an unusual presentation of (metastatic) anaplastic thyroid carcinoma and concomitant papillary thyroid cancer metastasis within a contralateral lymph node. This was initially perceived as a left pyriform fossa mass involving and compressing her left hemi-larynx on clinical and radiological examination. Following the identification of BRAF V600E mutation on CGP, she was started on targeted therapy with the BRAF inhibitor dabrafenib and the MEK inhibitor trametinib and demonstrated excellent clinical and radiological response following 7 months of treatment. She has subsequently undergone total thyroidectomy alongside with bilateral neck dissection, and is due to start radioactive iodine treatment to reduce the risk of recurrence of disease.

## Clinical presentation

A 60-year-old female presented to outpatient clinic (ENT) with complaints of sore throat, progressive dysphonia and dysphagia to solids, alongside with frequent choking and aspiration episodes over a 6-month period. The patient further reported left-sided otalgia, and weight loss. She denied any dyspnoea. There was no previous history of cancer within the family. Prior medical history was non-significant.

A fine nasal endoscopy was performed in clinic, which revealed a left pyriform fossa mass involving and compressing her left hemi-larynx with the larynx being fixed. The airway was significantly narrowed and compromised, but the patient had no stridor at the time of assessment. The neck laryngeal framework appeared broadened with loss of the laryngeal click.

## Investigations

MRI of the neck was performed demonstrating a large necrotic mass lesion which appeared to be centred over the left pyriform fossa. This extended superiorly along the left posterolateral pharyngeal wall to the level of the epiglottic tip, and inferiorly demonstrated invasion of the left glottis extending to the level of the left superior thyroid lobe with involvement of the left thyrohyoid and sternothyroid strap muscles. Medially, the left aryepiglottic fold was also involved with significant reduction in supraglottic airway calibre and there was posterior extension of the mass into the retrovisceral space crossing the midline to the right side, abutting the medial aspect of the right carotid sheath. The left thyroid lamina was invaded.

**Figure 1.**
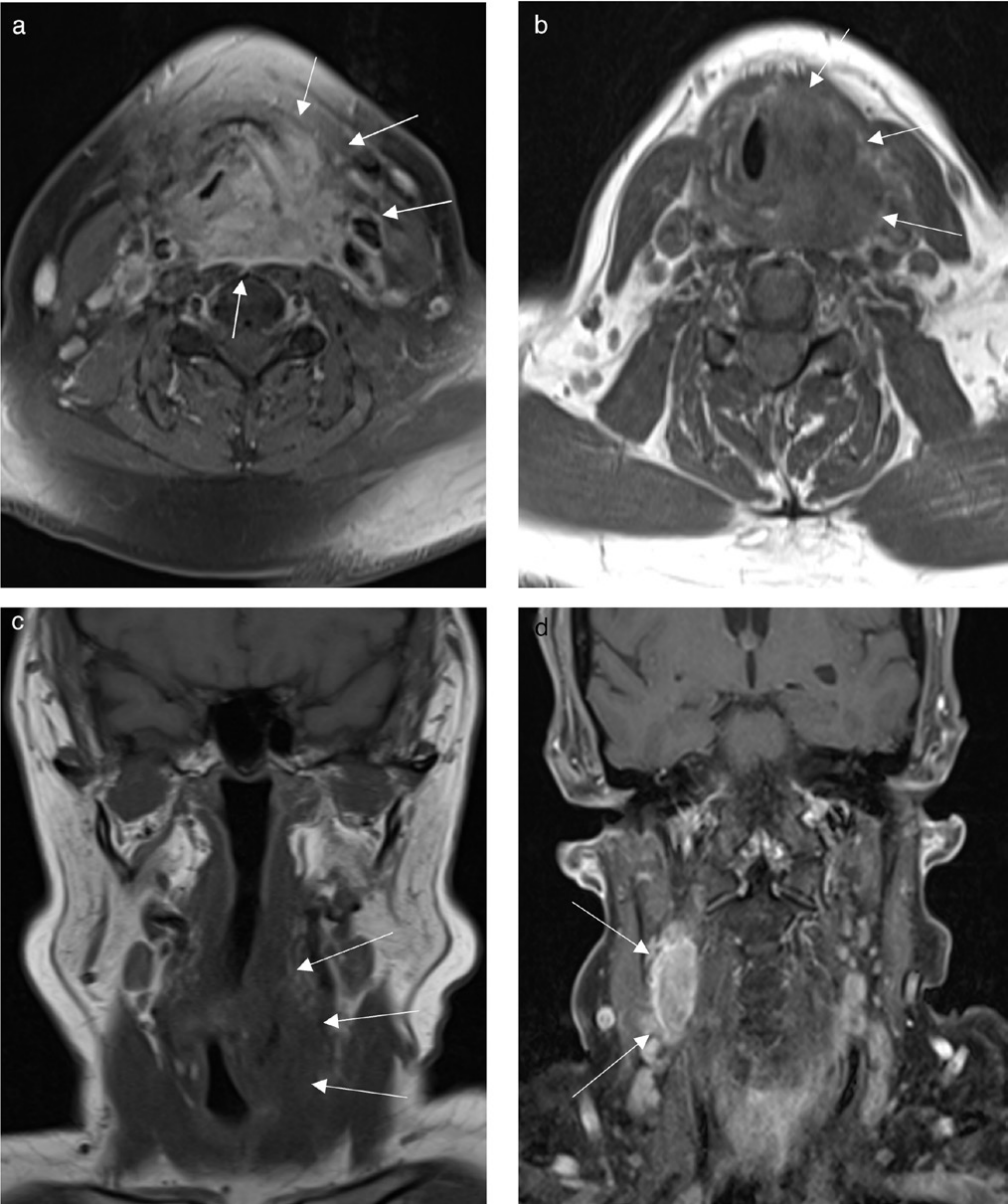
(a) Axial MRI fat saturated T1 post-contrast image shows an enhancing infiltrative mass involving the left glottis which appears thickened and displaced to the right side. This abuts the left carotid sheath, extending posteriorly into the retrovisceral space and crosses the midline to the right side (arrows). (b) Axial MRI T1 image shows infiltration of the left sternohyoid muscle and anterior cervical space. (c) Coronal MRI T1 image shows extension along the posterolateral hypopharyngeal wall, inferiorly inseparable from the left superior thyroid lobe. (d) Coronal MRI fat saturated T1 post-contrast image shows an enlarged enhancing right level two lymph node.

There were associated pathological cervical lymph nodes noted at bilateral level II levels, left level III, and left level IV levels. These demonstrated a rounded outline, heterogeneous STIR signal and enhancement concerning for metastases radiologically (Figure 1). At this stage, there was suspicion for a primary hypopharyngeal mucosal malignancy centred on the left pyriform fossa, with metastatic lymph nodes.

**Figure 2.**
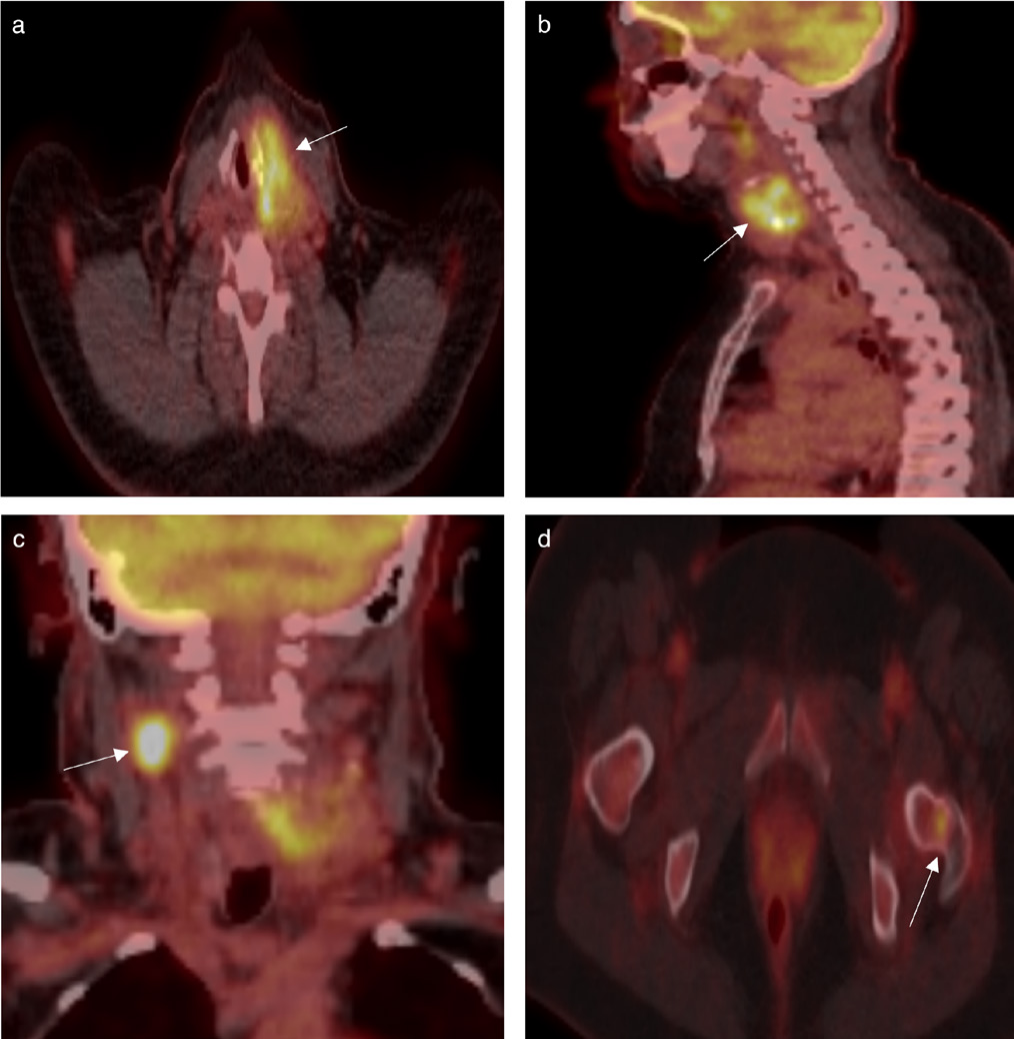
FDG PET scan CT axial (a) and sagittal (b) images confirm high grade metabolic activity extending along the left posterolateral hypopharyngeal wall indicating diffuse infiltration by the primary lesion. (c) FDG PET CT coronal image confirms high grade metabolic activity in a right level two lymph node indicative of nodal metastasis. (d) FDG PET CT axial image demonstrates focal increased metabolic activity in the left femoral neck raising suspicion for skeletal metastasis. FDG, fludeoxyglucose; PET, positron emission tomography.

A subsequent fludeoxyglucose positron emission tomography CT raised suspicion for bony metastasis, demonstrating focal high grade activity in the left femoral neck (Figure 2d) with sclerotic changes on the CT component of the examination. There was no evidence of pulmonary or solid organ metastases.

**Figure 3.**
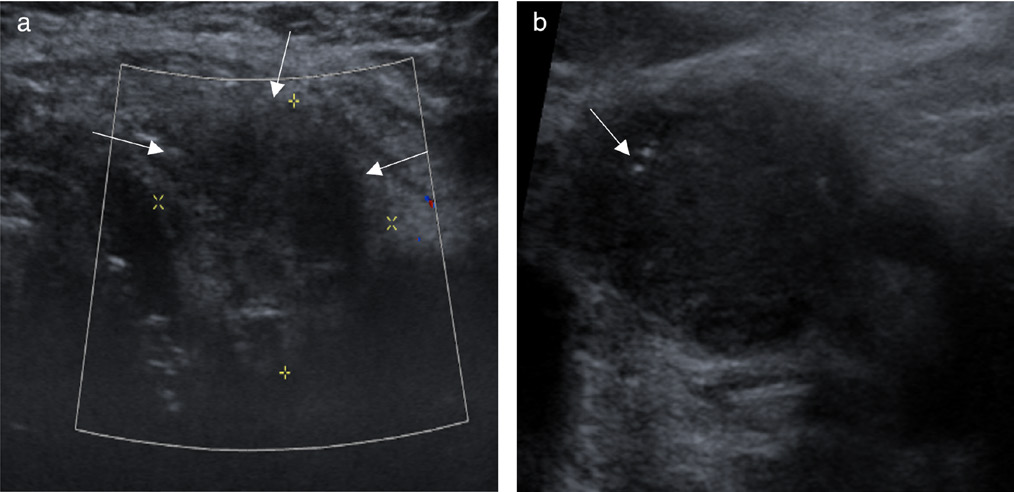
(a) Axial ultrasound image confirms an ill defined hypoechoic nodule in the left thyroid lobe (arrows). (b) Ultrasound confirms a morphologically abnormal hypoechoic lymph node with flecks of microcalcification within it (arrow).

Subsequent ultrasound scans were performed, which further demonstrated the primary lesion to be a large hypoechoic mass throughout the left side of the neck involving the upper pole of the thyroid, the adjacent strap muscles anteriorly (Figure 3a). This appear to be contiguous with the left hypopharynx and larynx. There was some acoustic shadowing indicative of coarse calcification within the lesion.

At this stage, this raised a new suspicion for a malignant primary other than squamous cell carcinoma, as calcification was considered an atypical feature.

There were multiple morphologically abnormal cervical lymph nodes throughout the neck, seen from left levels II-IV and at right level II. Sonographically, these lymph nodes appeared rounded and hypoechoic with a lack of distinct hilum and central coarse calcification (Figure 3b).

Based on these morphological ultrasound features, the epicentre of the primary lesion was re-assessed to be at the superior pole of the left thyroid lobe. Ultrasound-guided fine needle aspiration (FNA) of the primary lesion and pathological right level II lymph node were performed.

## Cytological findings

**Figure 4.**
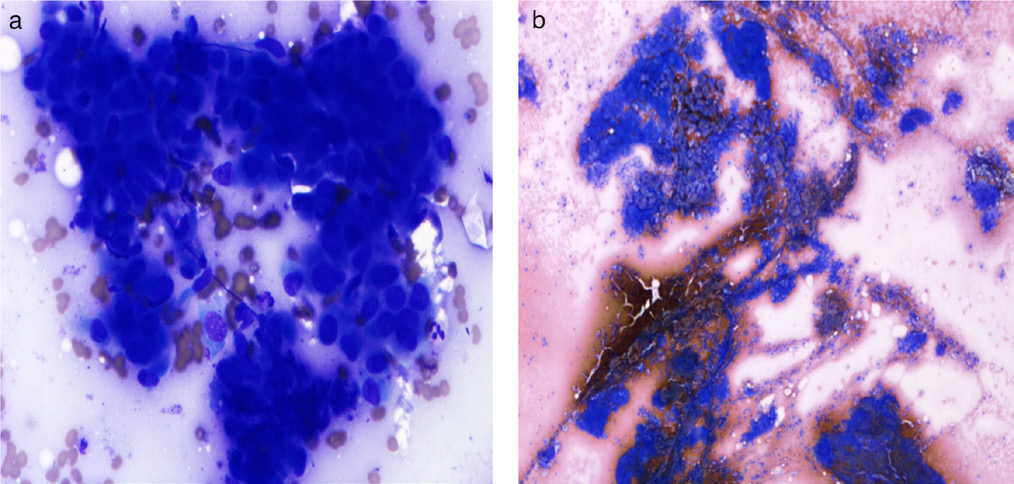
(a) Left hypopharyngeal mass: a cellular aspirate showing the features of a poorly differentiated malignant tumour. The malignant cells have high nuclear/cytoplasmic ratios and dense cytoplasm. Their nuclei are enlarged and hyperchromatic and contain single to multiple nucleoli with occasional nuclear inclusions. (b) Right level II lymph node: a cellular aspirate showing the features of a poorly differentiated malignant tumour seen in sheets, papillaroid clusters and caps. The malignant epithelial cells have scant to moderate amounts of cytoplasm and enlarged round to oval nuclei containing fine, granular chromatin and micronucleoli. Intranuclear inclusions, longitudinal nuclear grooves and psammoma bodies were identified. Occasional cells had dense cytoplasm (squamoid metaplasia). (a) and (b): Direct spreads, May Grunwald Giemsa (MGG).

Rapid onsite evaluation (ROSE) was performed on FNAs from the left parapharyngeal mass and the right level II lymph node. Both revealed a poorly differentiated, malignant tumour (Figure 4a and b).

Formal cytological assessment showed the left hypopharyngeal mass to be consistent with the squamoid variant of anaplastic thyroid carcinoma. On immunohistochemistry, the tumour stained positively with CK7, CK19, p40, CK5/6 and PAX-8. TTF-1, HBME-1, CK20 and calcitonin were negative.

The right level II lymph node showed features of papillary thyroid carcinoma. This tumour stained positively for CK7, CK19, PAX8 and TTF-1. There was patchy staining with thyroglobulin and p40 and very occasional staining with HBME-1. The tumour was negative for CK20, CK5/6 and calcitonin.

Sections from the agar cell block of the left hypopharyngeal mass aspirate were sent for BRAF V600E mutation analysis. A BRAF V600E mutation was detected which suggested that the anaplastic thyroid carcinoma most likely arose from a pre-existing papillary thyroid carcinoma.

The diagnosis was confirmed on core needle biopsy.

With a large left necrotic thyroid mass and metastatic contralateral nodal involvement identified from FNA and core biopsy specimens, a diagnosis of anaplastic thyroid cancer (pT4a pN1b M1) was established.

## Differential diagnosis

Considering the patient’s symptoms of dysphonia in conjunction with the fine nasal endoscopy findings of a left pyriform fossa soft tissue mass invading the left hemi-larynx, a primary hypopharyngeal or laryngeal malignancy should also be considered within the differential diagnosis.

### Outcome and follow-up

Following the identification of BRAF V600E mutation on CGP from core biopsy specimens, the patient was initiated on targeted therapy with the BRAF inhibitor dabrafenib and the MEK inhibitor trametinib.

The patient was subsequently reviewed in clinic 2 months after the initiation of targeted therapy, and reported clinical response to treatment with improvement of her symptoms of dysphonia and dysphagia. On clinical examination, the palpable mass on the left side of her neck had markedly decreased in size.

A restaging CT performed 3 months after the initiation of treatment demonstrated an excellent radiological response to treatment. There was a significant reduction in size of the diffusely infiltrating left-sided neck mass. The pathological cervical lymph nodes also demonstrated a reduction in size.

## Treatment

**Figure 5.**
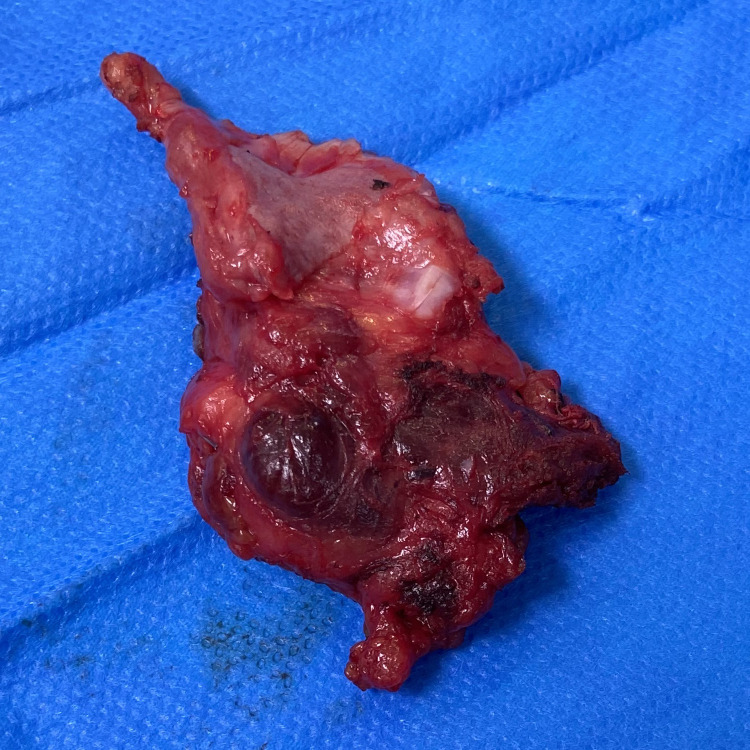
The pathological findings of the left hemithyroidectomy surgical specimen demonstrated an area of follicular variant papillary thyroid carcinoma present within the left hemithyroid, with widespread differentiated carcinoma (follicular architecture) present within the associated scar tissue. These areas represented residual foci of follicular variant papillary thyroid carcinoma within the treated area of anaplastic carcinoma. It is likely that the anaplastic carcinoma represented a dedifferentiated papillary thyroid carcinoma.

The patient has subsequently undergone left hemithyroidectomy (Figure 5) and left neck dissection, followed by completion right hemithyroidectomy and right neck dissection after 6 months. A completion thyroidectomy was performed in order to reduce the risk of recurrence of disease and facilitate radioactive iodine treatment.

A restaging fludeoxyglucose PET CT scan performed 3 months following the initial surgery of left hemithyroidectomy and left neck dissection has demonstrated excellent radiological response to treatment following immunotherapy and surgery. She is currently under clinical and radiological surveillance

## Discussion

Anaplastic thyroid carcinoma (ATC) is considered a rare and aggressive form of thyroid cancer. It has been proposed that ATC develops by dedifferentiation in pre-existing well-differentiated carcinomas although the sequence of tumorogenesis remains unclear. ATC can also present concomitantly with differentiated thyroid cancers in 5–17% of patients. A study by Nel et al demonstrated that out of 82 patients, 5% were found to have the co-existence of papillary thyroid cancer. A few cases have been described in literature in which this transformation occurs at a metastatic site, *e.g.* within a cervical lymph node.

Most patients may present with clinical symptoms of neck swelling, dyspnoea, dysphagia and dysphonia.

The objectives of this report, are to highlight the unusual presenting features of this aggressive thyroid malignancy as a mass centred within the hypopharynx both on initial clinical examination and MRI. This highlights the importance of further correlation with ultrasound images and pathological specimens as part of further assessment and staging for patients on the head and neck cancer pathway.

In terms of management, this case presents the favourable treatment outcomes when a therapeutic regimen was guided by mutation analysis. Given the low incidence of such cases, the optimal treatment regimen is still an area of interest within research. The treatment selection of the patient was based on the presence of the BRAF V600E mutation. Despite the presence of metastatic lymph nodes and distant metastatic disease, the patient demonstrated excellent clinical and radiological response to treatment within 3 months of commencement of targeted therapy with the BRAF inhibitor dabrafenib and the MEK inhibitor trametinib.

Overall, this highlights that future research into advanced treatment options including targeted therapy and/or immunotherapy for both DTC and ATC should continue. The patient has subsequently undergone hemithyroidectomy alongside with selective left neck dissection. The patient is due to undergo treatment and is currently under clinical and radiological surveillance.

## Learning points

- Anaplastic thyroid cancer can present as a diagnostic challenge due its aggressive nature and involvement of adjacent anatomical structures. Patients may present with symptoms of dysphagia and dysphonia before presenting with a palpable neck mass. Therefore, it is important for this to be considered within the differential for a patient with these symptoms.
- Multimodality imaging and histopathological diagnosis is essential for accurate tumour assessment, staging and guiding treatment which must be implemented rapidly due to the aggressive nature of this malignancy and associated poor prognosis.
- The focus of treatment has shifted to the development of novel therapies based on the availability of CGP. The combination of dabrafenib/trametinib has showed incredible efficacy in patients with locally advanced or metastatic BRAF V600E-mutated ATC. Hence, BRAF mutation should be obtained on all patients with ATC at diagnosis.
